# Supplementary material for: A Regulatory-Compliant Genotoxicity Study of a Mixture of C60 and C70 Fullerenes Dissolved in Olive Oil Using the Mammalian Micronucleus Test
Source: Nanomaterials (Basel). 2025 Jun 5;15(11):870. doi: 10.3390/nano15110870 (PMC12156957; doi:10.3390/nano15110870)
Supplement: Supplementary file 1 [file nanomaterials-15-00870-s001.zip › nanomaterials-3682683-supplementary.pdf]

Supplementary information to:

A Regulatory-Compliant Genotoxicity Study of a Mixture of C60 and C70 Fullerenes Dissolved in Olive Oil Using the Mammalian Micronucleus Test

Fathi Moussa

Institut de Chimie Physique, CNRS–UMR 8000, Paris–Saclay University,

91400 Orasay, France; fathi.moussa@universite-paris-saclay.fr

Preamble: This supplementary information is the original official report of the certified European Laboratory that carried out the entire study. This report includes the details of the experimental procedures and all raw data as well as the legal attestations related to the study.

The test item ESS UTHXX in Organic Extra Virgin Olive Oil refers to the mixture of fullerenes (C60/C70; 4.1/1, w/w) dissolved in Extra Virgin Olive Oil (Appendix VI, Certificate of analysis).

TC

TOXI-COOP ZRT.

TOXI-COOP ZRT

Address: 8230 Balatonfüred, Arácsi út 97
Phone: +36-30-678-2994

Final Report

ESS UTHXX in Organic Extra Virgin Olive Oil
MOUSE MICRONUCLEUS TEST

(Report including Appendices total pages 28)

Study no: **842-474-6963**

Date of Final Report: **November 24, 2022**

Sponsor:

SES Research Inc.
5999 West 34th Street
Suite 106
Houston, TX 77079
USA

Author:

Dr. Erzsébet Béres
Toxi-Coop ZRT.
Arácsi út 97
8230 Balatonfüred,
Hungary

This page was intentionally left blank for statements by the Sponsor or submitter.

Table of Contents Page

Statement of the Study Director **6**
Statement of the Management **7**
Quality Assurance Statement **8**
General Statements and Responsibilities **9**
Experimental Schedule **10**
1.0 Summary **11**
2.0 Study Objective and Introduction **12**
3.0 Regulatory Guidelines and Test Methods **12**
4.0 Archiving **12**
5.0 Materials and Methods **13**
5.1 Test Item 13
5.1.1 Name and Data of Test Item 13
5.1.2 Identification, Receipt 13
5.1.3 Formulation of Test and Positive Control Items 13
5.2 Positive and Negative Controls 13
5.2.1 Positive Control 13
5.2.2 Negative Control 14
5.2.3 Other Chemicals Used in the Experiment 14
5.3 Test System 14
5.3.1 Experimental Animals 14
5.3.2 Husbandry 15
5.3.3 Food and Feeding 15
5.3.4 Water Supply 15
5.3.5 Identification and Randomization 15
6.0 Description of the Test Procedure **16**
6.1 Preliminary experiments 16
6.1.1 Preliminary Solubility Test 16
6.1.2 Preliminary Toxicity test 16
6.2 Micronucleus Test 16
6.2.1 Doses 16
6.2.2 Application, Clinical Examination and Sampling Times 17
6.2.3 Bone Marrow Preparation and Staining 17
6.2.4 Examination of Slides 17
6.2.5 Acceptability of the Test 18
6.2.6 Evaluation of Experimental Data 18
6.3 The Permission of the Institutional Animal Care and Use Committee (IACUC) 19
7.0 Deviations **19**
7.1 Deviation to the Study Plan 19
7.2 Deviation to the Guideline 19
8.0 Amendment Procedure **19**
9.0 Results and Discussion **20**
10.0 References **22**
APPENDICES **23**
APPENDIX I: ESS UTHXX in Organic Extra Virgin Olive Oil MOUSE MICRONUCLEUS TEST SUMMARY TABLE **24**

APPENDIX II: ESS UTHXX in Organic Extra Virgin Olive Oil MOUSE MICRONUCLEUS TEST INDIVIDUAL DATA.......... 25
APPENDIX III: MOUSE MICRONUCLEUS TEST HISTORICAL CONTROL DATA.......... 26
APPENDIX IV: CONTENTS OF THE DIET SSNIFF® SM R/M-Z+H COMPLETE DIET FOR RATS AND MICE.......... 27
APPENDIX V: GLP CERTIFICATE OF TOXI-COOP ZRT.......... 28
APPENDIX VI: COPY OF THE CERTIFICATE OF ANALYSIS OF THE TEST ITEM.......... 29

The following prints of this report are issued:

Paper prints:
Original 1 of 2 — Archived at TOXI-COOP ZRT.
Original 2 of 2 — Released to the sponsor

Electronic copy:
Electronic copy 1 of 1 — An electronic copy in PDF format is released to the sponsor.

The electronic file is an unaudited copy, generated after finalization of the report. The sponsor is reminded that PDF files are not sufficiently protected against modification. Therefore, TOXI-COOP ZRT. cannot take any responsibility for the content of the electronic copy. The sponsor is using the electronic copy on his own responsibility.

Statement of the Study Director

This study has been performed in accordance with the study plan, the OECD Guidelines for Testing of Chemicals No.: 474 (2016), EPA Health Effects Test Guidelines, OPPTS 870.5395 and the Principles of Good L boratory Practice (Hungarian GLP Regulations: 42/2014 (VIII. 19.) EMMI decree of the Minister of Human Capacities which corresponds to the OECD GLP, ENV/MC/CHEM (98)17).

J the undersigned declare that this report constitutes a true record of the actions undertaken and the results obtained in the course of this study.

Signature: [illegible]

Dr Erzsebet Beres

Date: November 24, 2022

Statement of the Management

According to the conditions of the research and development assignment between **SES Research Inc.** (Sponsor) and **TOXI-COOP ZRT** (as Test Facility)" ESS UTHXX in Organic Extra Virgin Olive Oil Mouse Micronucleus Test" has been performed in the laboratory of TOXI-COOP ZRT. as a GLP study.

Signature: ________________________________ Date: Nov 24, 2022

Dr. Gábor Hirka

Quality Assurance Statement

Study no: **842-474-6963**

Study Title: **ESS UTHXX in Organic Extra Virgin Olive Oil** Mouse Micronucleus Test

Test Item: **ESS UTHXX in Organic Extra Virgin Olive Oil**

This study has been inspected and this report audited by the Quality Assurance Unit in compliance with the Principles of Good Laboratory Practice. As far as it can be reasonably established the methods described and the results incorporated in this report accurately reflect the raw data generated in the course of this study.

All inspections, data reviews and the report audit were reported in writing to the study director and to the management. The dates of such inspections and of the report audit are given below:

Date	Inspection/Audit	Date of report to Management	Date of report to Study Director
August 31, 2022	Study Plan	August 31, 2022	August 31, 2022
October 19, 2022	Microscopic evaluation	October 19, 2022	October 19, 2022
November 02, 2022	Draft Report	November 02, 2022	November 02, 2022
November 24, 2022	Final Report	November 24, 2022	November 24, 2022

Signature: [illegible]
11 rmann
Quality Assurance Unit

Date: Nov. 24, 2022

General Statements and Responsibilities

**Study title:**	**ESS UTHXX in Organic Extra Virgin Olive Oil Mouse Micronucleus Test**
**Study number:**	**842-474-6963**
**Sponsor:**	**SES Research Inc.**<br>5999 West 34th Street<br>Suite 106<br>Houston, TX 77079<br>USA<br>Phone: +1 (800)275-0082
**Sponsor's scientific monitor:**	**John R. Endres**<br>Chief Scientific Officer<br>AIBMR Life Sciences, Inc.<br>1425 Broadway, Suite 458<br>Seattle, WA 98122<br>(254) 286-2888<br>www.aibmr.com | @AIBMRinc
**Test facility:**	**TOXI-COOP ZRT.**<br>8230 Balatonfüred, Arácsi út 97<br>(1103 Budapest, Cserkesz u. 90)<br>Hungary<br>Phone: +36-30-678-2994
**Location of study:**	**TOXI-COOP ZRT.**<br>8230 Balatonfüred, Ady utca12, Arácsi út 97
**Test facility management:**	**Dr. Gábor Hirka**
**Study director:**	**Dr. Erzsébet Béres**
**Quality assurance unit:**	**Ildikó Hermann**
**Responsible technical assistant:**	Zsuzsanna Frank – technical assistant<br>Jurácsikné Sereg Kornélia – technician<br>Szabóné Oláh Mónika – technician<br>Márta Tenk – statistical data processing<br>Pátkai Zsófia – technician<br>Oravecz Viktória – technician<br>Hummel Zsolt – technician<br>Horváth Atilla – technician<br>Németh Dániel – technician<br>Gaál Zoltán – animal house technical manager<br>Klucsik Anita – technician<br>Küronya Bence – technician

Experimental Schedule

**Start of preliminary toxicity test:**	August 16, 2022
**Start of Main Experiment:**	October 04, 2022
**Animal receipt:**	September 28, 2022
**Animal identification:**	October 03, 2022
**Randomisation:**	October 03, 2022
**The day of treatment:**	October 04 and 05, 2022
**End of in-life phase:**	October 06, 2022
**End of examination:**	October 20, 2022
**Date of Draft Report:**	October 28, 2022
**Date of Final Report:**	November 24, 2022

1.0 Summary

The potential mutagenic activity of ESS UTHXX in Organic Extra Virgin Olive Oil was examined in bone marrow of male NMRI mice.

The doses of the test item for the Micronucleus Test were determined according to a preliminary oral toxicity study. The doses selected were 500, 1000 and 2000 mg ESS UTHXX in Organic Extra Virgin Olive Oil per kg body weight.

Negative (vehicle) control and a positive control group were included. Treatment was carried out in Helianthii Annui Oleum Raffinatum with a constant treatment volume (10 mL/kg body weight). The test item and negative (vehicle) control item were administered by gavage two times at 24-hour intervals. Cyclophosphamide dissolved in Aqua ad injectabilia (positive control) and was administered once, intraperitoneally with a treatment volume of 10 mL/kg body weight. In the low, mid and high dose groups and solvent control group the sampling was made once at 24 hours after the second treatment. In animals treated with Cyclophosphamide (60 mg/kg bw.), the sampling was performed only at 24 hours post-treatment. Five animals per dose groups were used. Four thousand polychromatic erythrocytes (PCEs) were scored per animal to assess the micronucleated cells.

The two times oral administration of 500 mg/kg body weight, 1000 mg/kg body weight and 2000 mg/kg body weight of ESS UTHXX in Organic Extra Virgin Olive did not induce increases in the frequency of MPCEs in male mice at 24 hours after the second treatment compared to the negative and to the historical control groups.

The proportion of immature among total (immature + mature) erythrocytes was determined for each animal by counting a total of at least 500 erythrocytes. Compared to the negative and historical control groups the number of polychromatic erythrocytes (PCEs) at 24 hours after the second treatment in the dose group of 500 mg/kg body weight was not affected. Compared to the negative and historical control groups the reduction in PCE/total erythrocytes ratio at 24 hours after the second treatment were statistically lower in the dose groups of 1000 and 2000 mg/kg body weight. In the dose group of 2000 mg/kg body weight, the reduction in PCE/total erythrocytes ratio is considered to be biologically significant. This is considered to be relevant finding to exposure of the test item and indicate that the test item or its degradation products reached the bone marrow and caused toxicity there.

The frequencies of micronucleated polychromatic erythrocytes (MPCEs) for the negative and positive control mice were within acceptable ranges and compatible with the historical control data for this laboratory. Cyclophosphamide treated mice (60 mg/kg body weight) showed a large, statistically significant increase in the MPCE number compared to the negative and historical controls. Thus, the study is considered valid.

No biologically and statistically significant increases in the frequency of MPCEs were seen in the groups of mice treated with ESS UTHXX in Organic Extra Virgin Olive Oil compared to the negative and to the historical control groups.
ESS UTHXX in Organic Extra Virgin Olive Oil did not show any genotoxic activity in this Mouse Micronucleus Test.

2.0 Study Objective and Introduction

The purpose of this study is to determine whether the test item causes genotoxic effects resulting in the formation of micronuclei in erythrocytes of treated animals.
The mammalian in vivo micronucleus test is used for the detection of damage induced by the test substance to the chromosomes or the mitotic apparatus of erythroblasts by analysis of erythrocytes as sampled in bone marrow cells of animals, usually rodents. The purpose of the micronucleus test is to identify substances that cause cytogenetic damage which results in the formation of micronuclei containing lagging chromosome fragments or whole chromosomes. When a bone marrow erythroblast develops into a polychromatic erythrocyte, the main nucleus is extruded; any micronucleus that has been formed may remain behind in the otherwise anucleated cytoplasm. Visualisation of micronuclei is facilitated in these cells because they lack a main nucleus. An increase in the frequency of micronucleated polychromatic erythrocytes in treated animals is an indication of induced chromosome damage.

3.0 Regulatory Guidelines and Test Methods

This study was performed in compliance with the procedures indicated by the following internationally accepted guidelines and recommendations:

- Ninth Addendum to OECD Guidelines for Testing of Chemicals, Section 4, No. 474, ″Mammalian Erythrocyte Micronucleus Test.″ adopted 29th July, 2016
- EPA Health Effects Test Guidelines, OPPTS 870.5395 "Mammalian Erythrocyte Micronucleus Test" August-1998.

4.0 Archiving

The study documents and samples as listed below will be archived according to the OECD GLP and to the Toxi-Coop Zrt. SOPs in the archives of TOXI-COOP Zrt. (H-8230 Balatonfüred, Galamb u. 12/A., Hungary):

- Study plan and any amendments (15 years)
- All raw data (15 years)
- Retained sample of the test item (5 years)
- Retained sample of the positive control reference items (5 years)
- Correspondence (15 years)
- Study report and any amendments (15 years)
- Slides (12 years)

For the first 5 years archiving is included, thereafter archiving occurs at additional costs of the Sponsor. After this period, the Sponsor will be notified to decide on further archiving to comply with current legal requirements.
After the retention time all the archived materials listed above will be returned to the Sponsor or retained for a further period if agreed by a contract or destroyed on their behalf.
None of the above cited documents or material will be discarded without the explicit written consent of the Sponsor.
At the end of the study, any remaining test item will be returned to the Sponsor or will be discarded, unless otherwise instructed by the Sponsor.

5.0 Materials and Methods

5.1 Test Item

5.1.1 Name and Data of Test Item

Name:	ESS UTHXX in Organic Extra Virgin Olive Oil
Product Code:	ESS-P411
Lot#:	XUTH025
Appearance:	Brown Reddish, liquid
Odour:	Faint oil odour
Date of manufacture:	04/07/2022
Expiry date:	04/07/2024
Storage conditions:	At room temperature, protect from humidity

Information based on the Certificate of Analysis and correspondence with Sponsor's Scientific Monitor.

5.1.2 Identification, Receipt

The test item of a suitable chemical purity was supplied by the Sponsor. All precautions required in the handling and disposal of the test item is outlined by the Sponsor. These documents are part of the raw data. Identification of the test item was performed in the TOXI-COOP ZRT. on the basis of its appearance (Information included in the covering documentations, which was supplied by the Sponsor).

5.1.3 Formulation of Test and Positive Control Items

The test item was dissolved in Helianthii Annui Oleum Raffinatum for the treatment. The necessary amount of test item was weighed into a calibrated volumetric flask. A partial volume of Helianthii Annui Oleum Raffinatum was added and the formulation was stirred until homogeneity is reached. The test item was used for treatment in concentrations of 50 mg/mL, 100 mg/mL and 200 mg/mL prepared with Helianthii Annui Oleum Raffinatum. The formulations were prepared fresh on day of dosing and used within 15 minutes.
No formulation analysis was performed.
Cyclophosphamide (positive control) was dissolved in Aqua ad injectabilia for treatment.

5.2 Positive and Negative Controls

Positive and negative (solvent) controls were included in the experiments.

5.2.1 Positive Control

Name:	Cyclophosphamide
CAS No.:	6055-19-2
Batch number:	MKCN3646
Supplier:	Sigma-Aldrich St. Louis, MO 63103 USA
Retest date:	October 2023
Storage condition:	At 2-8°C

5.2.2 Negative Control

Vehicle of test item

Name:	Helianthii Annui Oleum Raffinatum
Batch No.:	8008219001
Supplier:	MAGILAB KFT, Hungary
Expiry:	November 30, 2022
Storage condition:	at room temperature

Solvent of positive control item

Name:	Aqua ad injectabilia
Batch No.:	38461121
Supplier:	MAGILAB KFT, Hungary
Expiry:	November 30, 2022
Storage condition:	Do not store above 25 ° C

5.2.3 Other Chemicals Used in the Experiment

Chemical	Supplier / Manufacturer	Batch / Lot Number	Expiry / Retest Date
Foetal bovine serum	Sigma-Aldrich St. Louis, MO 63103 USA	0001655439	July 2026
Giemsa	MERCK KGaA Darmstadt, Germany	HX20201004	September 30, 2025
Aqua purificata	MAGILAB KFT: Hungary	2205-8253	November 10, 2022
Methanol	lach:ner Czech Republic. Neratovice.	PP/2022/03527	March 31, 2025
E-Z Mount™	epredia Enhancing precision cancer diaggnpostics Kalamazoo, MI 49008, USA	527711	March 2024

5.3 Test System

5.3.1 Experimental Animals

Species and strain:	Win: NMRI mice
Source:	TOXI-COOP ZRT<br>Budapest, HUNGARY
Hygienic level at arrival:	SPF (Specific Pathogen Free)
Hygienic level during the study:	good conventional
Justification of strain:	The NMRI mouse is one of the standard animals used internationally in this type of mutagenicity testing.
Number of animals:	Pretest: 2 males and 2 females / dose groups<br>Main test: 25+2 males (two additional male mice were dosed in highest group)<br>5 male animals/group<br>(7 male animals in the high dose group), 5 groups
Age of animals:	8 weeks
Body weight:	Body weight was recorded for all animals at randomization and prior to dosing.
Body weight range<br>At starting of the treatment:	32,2 – 37.8 g
Acclimatization time:	6 days

5.3.2 Husbandry

Animal health:	Only animals in acceptable health condition were used for the test.
Housing:	Group caging (2 animal/cage in the pre-test and in the high dose group of main tests and 5 animals/cage in the other groups of the main test).
Cage type:	I. type polypropylene/polycarbonate
Bedding:	laboratory bedding
Light:	12 hours daily, from 6.00 a.m. to 6.00 p.m.
Temperature:	22 ± 3 °C
Relative humidity:	40 - 70 %
Housing/Enrichment:	Rodents are group-housed to allow social interaction, and with deep wood sawdust bedding, to allow digging and other normal rodent activities.

5.3.3 Food and Feeding

The animals received pellet diet (ssniff® SR/M-Z+H) produced by ssniff Spezialdiäten GmbH (Experimental Animal Diets Inc., 59494 Soest, Germany) ad libitum. Diet composition is presented in APPENDIX IV. Consumption was controlled visually on a daily base.

5.3.4 Water Supply

The animals received water, as for human consumption, ad libitum, from 250 mL bottles.

5.3.5 Identification and Randomization

The individual identification of the animals was performed by numbers on the tail. The cages were marked with identification cards, with information about cage number, study code, sex, dose group and individual animal numbers and species and strain of animal. The animals were randomly assigned to control and test groups using a randomization scheme. The randomization was checked according to the actual body weights verifying the homogeneity and deviations between the groups.

6.0 Description of the Test Procedure

6.1 Preliminary experiments

6.1.1 Preliminary Solubility Test

A non GLP Preliminary Solubility Test was performed August 04, 2022. The test item was formulated in Helianthii Annui Oleum Raffinatum. Homogeneous formulation was obtained up to a concentration of 200 mg/mL.

6.1.2 Preliminary Toxicity test

Justification of the doses

A non GLP Preliminary toxicity test was performed (August 16-18, 2022) to identify the appropriate maximum dose level for the main test as no toxicity data in mice was available. The preliminary toxicity test determined the MTD based on death and clinical signs of test item related toxicity and whether there are differences in toxicity between the male and female animals. Groups of two male and female mice were treated two times at 24-hour intervals by oral gavage at dose level of 2000 mg/kg bw. The treatment was carried out in Helianthii Annui Oleum Raffinatum with a constant volume (10 mL/kg bw). Animals were examined regularly for toxic signs and mortalities.

On the basis of the results of this preliminary toxicity test, the following doses were selected for the Mouse Micronucleus Test: 500, 1000 and 2000 mg/kg body weight. Please find details in section 9.0 Results and Discussion.

6.2 Micronucleus Test

6.2.1 Doses

The main test was performed using male mice because the toxic effect of the test item was similar in both sexes in the preliminary acute oral toxicity test.

The animals in the Mouse Micronucleus Test were treated according to scheme shown below:

Group	Treatment mg/kg bw	Dose concentration (mg/ mL)	No. of Analysable * Animals	Sampling time (after the last treatment)
1. Negative control	Solvent	0.0	5 Males	24 hours
2. Test item, low dose	500	50	5 Males	24 hours
3. Test item, mid dose	1000	100	5 Males	24 hours
4. Test item, high dose	2000	200	5 Males	24 hours
5. Positive, control	Cyclophophamide: 60	6.0	5 Males	24 hours

* Two additional male mice were dosed in the highest test item treated group to replace any which die before the scheduled sacrifice time. No deaths occurred in the original population (5 animals). Bone marrow smears were not prepared from the additional mice as they were not used as replacements although clinical signs were recorded and reported.

6.2.2 Application, Clinical Examination and Sampling Times

The test/solvent items were administered orally by gavage two times at 24-hour intervals.
The treatment volume was 10 mL/kg body weight. In the low, mid and high dose groups and the vehicle control group the sampling was made once at 24 hours after the second treatment. Five Male animals per dose group were used for sampling. Cyclophosphamide (positive control) was administered intraperitoneally with a treatment volume: 10 mL/kg body weight. Sampling was performed 24 hours after the beginning of the treatment and five male animals were used for sampling. The mice were examined regularly for visible signs of reactions to treatment, immediately after dosing, and periodically until sacrifice.

6.2.3 Bone Marrow Preparation and Staining

Bone marrow was obtained from two exposed femurs of the mice from every time point immediately after sacrificing (cervical dislocation). The bone marrow was flushed with foetal bovine serum (5 mL). After vortex mixing, the cell suspension was concentrated by centrifugation and the supernatant was discarded. Smears of the cell pellet were made on standard microscope slides. Slides were then dried at room temperature.

Subsequently the slides were stained as follows:

Fixed for a minimum of 5 minutes in methanol and allowed to air-dry.
Stained with Giemsa (10%) solution for 25 minutes.
Rinsing in distilled water.
Drying at room temperature (at least 12 hours).
Coating with Micromount.

6.2.4 Examination of Slides

Prior to microscopic analysis, one slide from each animal was given a code number for blind microscopic analysis. The code labels were covered the original animal numbers to ensure that the slides are scored without bias. Four thousand polychromatic erythrocytes (PCEs) were scored per animal to assess the micronucleated cells.
The frequency of micronucleated cells was expressed as percent of micronucleated cells based on the first 4000 PCEs counted in the optic field.
The proportion of immature erythrocytes among total (immature + mature) erythrocytes was determined for each animal by counting a total of at least 500 erythrocytes.

<u>Criteria for Identification of Micronucleated Erythrocytes</u>

A micronucleus is defined in following way:

- A bluish mauve strongly coloured uniform circular particle in the cell.
- The particle should have a certain size and it should be located inside the cells.
- During focusing, the particle should stay uniform in colour /light refraction and shape within a large interval.
- Cells with two or more micronuclei were counted as single micronucleated cells.

6.2.5 Acceptability of the Test

The Micronucleus Test is considered acceptable if it meets the following criteria:

- The concurrent negative control data are considered acceptable for addition to the laboratory historical control database.
- The concurrent positive controls or scoring controls induce responses that are compatible with those generated in the historical positive control database and produce a statistically significant increase compared with the concurrent negative control.
- The appropriate number of doses and cells has been analysed.
- Each sampling time point of test item treated and control group should include at least 5 analysable animals.

6.2.6 Evaluation of Experimental Data

Statistics

Statistical analysis was done with SPSS PC+ software for the following data:

- The frequencies of micronucleated polychromatic erythrocytes in animals in the test and positive control groups were compared to the values found in the corresponding negative and historical control groups.
- The proportion of immature erythrocytes among total (immature + mature) erythrocytes in animals in the test and positive control groups were compared to the values found in the corresponding negative and historical control groups.
- The data was checked for a linear trend in mutant frequency with treatment dose using the adequate regression analysis by Microsoft Excel software.

Interpretation of Results

Providing that all acceptability criteria are fulfilled, the test item is considered clearly positive if:

- At least one of the treatment groups exhibits a statistically significant increase in the frequency of micronucleated immature erythrocytes compared with the concurrent negative control,
- This increase is dose-related at least at one sampling time when evaluated with an appropriate test, and
- Any of these results are outside the distribution of the historical negative control data (e.g., Poisson-based 95% control limits),
  (See Appendix III)

Providing that all acceptability criteria are fulfilled, the test item is considered clearly negative if the following criteria had been met:

- None of the treatment groups exhibits a statistically significant increase in the frequency of micronucleated immature erythrocytes compared with the concurrent negative control,
- There is no dose-related increase at any sampling time when evaluated by an appropriate test,

- All results are inside the distribution of the historical negative control data (e.g., Poisson-based 95% control limits),
- Bone marrow exposure to the test item occurred.

6.3 The Permission of the Institutional Animal Care and Use Committee (IACUC)

Institutional Animal Care and Use Committee (IACUC) of Toxi-Coop Zrt. permitted the conduct of the study.

7.0 Deviations

7.1 Deviation to the Study Plan

There was no deviation from the Study Plan.

7.2 Deviation to the Guideline

No deviations from testing guideline occurred.

8.0 Amendment Procedure

There was two Amendments (dated: August 31and October 03, 2022) to the Study Plan.

9.0 Results and Discussion

Preliminary Toxicity Test

A non GLP preliminary toxicity test (August 16-18, 2022) was performed to identify the appropriate maximum dose level for the main test.

Clinical signs and Mortality

Groups of two male and female mice were treated two times at 24-hour intervals by oral gavage at dose level of 2000 mg/kg body weight. The preliminary toxicity test also determined whether there are large differences in toxicity between the sexes. Animals were examined periodically for toxic signs and mortalities. No bone marrow smears were prepared from these mice.

The animals treated with the indicated dose level of ESS UTHXX in Organic Extra Virgin Olive Oil showed mortality rates as listed in the table below:

ESS UTHXX in Organic Extra Virgin Olive Oil mg/kg body weight	Number of Animals	Mortality
2000	2 Males and 2 Females	**0 males and 0 females**

In the male and female dose group of 2000 mg/kg no adverse reactions to treatment were observed

Micronucleus Test

Clinical signs and Mortality

The mice were examined for visible signs of reactions to treatment, immediately after dosing and periodically until sacrifice.

No animal died during the study.

No adverse reactions to treatment were observed in the mice of the negative and positive control groups.

No adverse reactions to treatment were observed **in the mice treated of 500 and 1000 and 2000 mg/kg.**

Two additional male mice were dosed in the highest (2000 mg/kg body weight) test item treated group to replace any which die before the scheduled sacrifice time. No death occurred in the original population (5 animals). Bone marrow smears were not prepared from the additional mice as they were not used as replacements.

Frequency of PCEs and MPCEs

A summary of the data and statistical analysis are presented in Appendix I. The proportion of immature erythrocytes among total (immature + mature) erythrocytes and the frequencies of polychromatic erythrocytes with micronuclei (MPCEs) for individual animals are shown in Appendix II.

The frequencies of micronucleated polychromatic erythrocytes (MPCEs) for the negative and positive control mice were compatible with the historical control data for this laboratory. Cyclophosphamide treated mice (60 mg/kg body weight) showed a large, statistically significant increase in the MPCE number compared to the negative and historical controls. Thus, the study is considered valid (See Appendix III).

Two times at 24-hour intervals oral administration of 500 mg/kg body weight 1000 mg/kg and 2000 mg/kg body weight of ESS UTHXX in Organic Extra Virgin Olive Oil did not induce increases in the frequency of MPCEs in male mice at 24 hours after the second treatment compared to the concurrent negative (solvent) and the historical control groups. The frequencies of micronucleated polychromatic erythrocytes (MPCEs) in the treated mice were within acceptable ranges and compatible with the historical control data for this laboratory.
The proportion of immature among total (immature + mature) erythrocytes was determined for each animal by counting a total of at least 500 erythrocytes. Compared to the negative and historical control groups the number of polychromatic erythrocytes (PCEs) at 24 hours after the second treatment in the dose group of 500 mg/kg body weight was not affected. Compared to the negative and historical control groups the reduction in PCE/total erythrocytes ratio at 24 hours after the second treatment were statistically lower in the dose groups of 1000 and 2000 mg/kg body weight. In the dose group of 2000 mg/kg body weight, the reduction in PCE/total erythrocytes ratio is considered to be biologically significant. This is considered to be relevant finding to exposure of the test item and indicate that the test item or its degradation products reached the bone marrow and caused toxicity there.

Conclusion

No biologically and statistically significant increases in the frequency of MPCEs were seen in the groups of mice treated with ESS UTHXX in Organic Extra Virgin Olive Oil compared to the negative and to the historical control groups.
ESS UTHXX in Organic Extra Virgin Olive Oil did not show any genotoxic activity in this Mouse Micronucleus Test.

10.0 References

1) Schmid, W. (1976). The micronucleus test for cytogenetic analysis. In Chemical Mutagens. Principles and Methods for their Detections, Vol. 4, ed. A. Hollander, Plenum, Press, New York, pp. 31-53.

2) Salamone, M.F., Heddle, J.A. (1983). The bone marrow micronucleus assay: rationale for a revised plan
In Chemical Mutagens., Principles and Methods for their Detections, Vol.8, ed. F.J.Serres. Plenum Press, New York, pp. 11-149.

3) Heddle, J.A., Hite, M. et al. (1983). The induction of micronuclei as a measure of genotoxicity.
A Report of the U.S. Environmental Protection Agency Gene-Tox Program, Mutation Research: 123, 61-118.

4) OECD Guideline for the Testing of Chemicals No. 474: Mammalian Erythrocyte Micronucleus Test. Adopted 29th July, 2016.

5) U.S. EPA: OPPTS 870.5395 Health Effects Test Guidelines: Mammalian Erythrocyte Micronucleus Test, August 1998

6) Hungarian Good Laboratory Practice Regulation: 42/2014 (VIII. 19.) EMMI decree of the Minister of Human Capacities which corresponds to the OECD GLP, ENV/MC/CHEM(98)17).

7) OECD Principles of Good Laboratory Practice, adopted by Council on 26th November 1997; Environment Directorate, Organization for Economic Cooperation and Development, Paris 1998.

APPENDICES

APPENDIX I:

ESS UTHXX in Organic Extra Virgin Olive Oil
MOUSE MICRONUCLEUS TEST
SUMMARY TABLE

Study Code: **842-474-6963** Test Item: **ESS UTHXX in Organic Extra Virgin Olive Oil**

Test System: **Win: NMRI mice** Sex: **Male**

No. of Animals: **5/group**

Groups	Sampling time (hour)	Total number of PCEs analysed	MPCE		PCE/ PCE+NCE	
			mean	± SD	mean	± SD
Negative Control	24	20000	**5.40**	1.14	0.52	0.02
500 mg/kg body weight	24	20000	**5.00**	0.71	0.52	0.01
1000 mg/kg body weight	24	20000	**5.60**	1.52	0.49**	0.01
2000 mg/kg body weight	24	20000	**5.40**	1.34	0.46**	0.01
Positive Control (60mg/kg body weight)	24	20000	**150.80*/****	9.86	0.37** DN	0.02

PCE = Polychromatic Erythrocyte
NCE = Normochromatic Erythrocyte
MPCE = Number of Micronucleated Polychromatic Erythrocyte referring to **4000PCE**
Positive Control = 60 mg/kg bw. Cyclophosphamide
Negative Control: Helianthii Annui Oleum Raffinatum
MPCE **:* = p < 0.05 to the vehicle control**
MPCE **:** = p < 0.01 to the historical control**
PCE/PCE+NCE:** **= p < 0.01 to the vehicle and historical control**
Kruskal-Wallis Non Parametric ANNOVA
U:Mann-Whitney U-test Versus Control
DN = Duncan's multiple range test

APPENDIX II:

ESS UTHXX in Organic Extra Virgin Olive Oil
MOUSE MICRONUCLEUS TEST INDIVIDUAL DATA

STUDY CODE : 842-474-6963
TEST ITEM : ESS UTHXX in Organic Extra Virgin Olive Oil
TEST SYSTEM : WIN: NMRI MICE

EXPERIMENTAL DATA OF MICRONUCLEUS STUDY **SEX: MALE**

SAMPLING TIME: 24th hours after the treatment

GROUPS	ANIMAL No.	BODY WEIGHT (g) 1st day	2nd day	APPL. VOLUME (ml) 1st day	2nd day	MPCE	PCE	NCE	PCE/ PCE+NCE
Vehicle Control	1	33,1	33,3	0,33	0,33	7	263	237	0,53
	2	35,1	36,2	0,35	0,36	6	270	230	0,54
	4	34,1	34,3	0,34	0,34	4	244	256	0,49
	5	34,1	33,7	0,34	0,34	5	263	237	0,53
	6	34,7	35,4	0,35	0,35	5	267	233	0,53
	MEAN	34,22	34,58	-	-	5,40	-	-	0,52
	±SD	0,76	1,20	-	-	1,14	-	-	0,02
500 mg/kg bw Test item	7	34,2	34,3	0,34	0,34	5	265	235	0,53
	8	34,7	34,8	0,35	0,35	5	260	240	0,52
	10	33,0	33,0	0,33	0,33	6	258	242	0,52
	11	33,9	33,8	0,34	0,34	5	264	236	0,53
	22	35,1	36,7	0,35	0,37	4	261	239	0,52
	MEAN	34,18	34,52	-	-	5,00	-	-	0,52
	±SD	0,80	1,39	-	-	0,71	-	-	0,01
1000 mg/kg bw Test item	13	33,9	35,4	0,34	0,35	7	250	250	0,50
	16	32,2	33,4	0,32	0,33	4	243	257	0,49
	21	35,7	36,6	0,36	0,37	4	239	261	0,48
	24	34,5	35,7	0,35	0,36	6	244	256	0,49
	34	34,7	35,5	0,35	0,36	7	245	255	0,49
	MEAN	34,20	35,32	-	-	5,60	-	-	0,49
	±SD	1,29	1,17	-	-	1,52	-	-	0,01
2000 mg/kg bw Test item	12	37,2	36,9	0,37	0,37	4	232	268	0,46
	15	33,0	33,1	0,33	0,33	6	234	266	0,47
	18	33,9	33,6	0,34	0,34	7	230	270	0,46
	19	35,5	34,9	0,36	0,35	4	229	271	0,46
	20	35,5	36,2	0,36	0,36	6	221	279	0,44
	27	-	-	-	-	-	-	-	-
	31	-	-	-	-	-	-	-	-
	MEAN	35,02	34,94	-	-	5,40	-	-	0,46
	±SD	1,62	1,63	-	-	1,34	-	-	0,01
Positive Control (Cyclophosphamid) 60 mg/kg	9	-	35,6	-	0,36	136	168	332	0,34
	28	-	35,4	-	0,35	146	186	314	0,37
	29	-	37,8	-	0,38	160	184	316	0,37
	32	-	33,5	-	0,34	154	192	308	0,38
	33	-	34,1	-	0,34	158	197	303	0,39
	MEAN	-	35,28	-	-	150,80	-	-	0,37
	±SD	-	1,66	-	-	9,86	-	-	0,02

Remarks:
PCE = Polychromatic Erythrocyte
NCE = Normochromatic Erythrocyte
MPCE = Number of Micronucleated Polychromatic Erythrocytes per 4000 PCE
- = no data

APPENDIX III:

MOUSE MICRONUCLEUS TEST
HISTORICAL CONTROL DATA

-	Number of Micronucleated Polychromatic Erythrocytes referring to counts of 4000 PCE		PCE/PCE+NCE	
	negative control	positive control (Cyclophosphamide)	negative control	positive control (Cyclophosphamide)
	Sampling time: 24 hours after the second treatment	Sampling time: 24 hours after the treatment	Sampling time: 24 hours after the second treatment	Sampling time: 24 hours after the treatment
**Mean**	**5.47**	**136.91**	**0.526**	**0.371**
**SD**	**1.08**	**10.21**	**0.010**	**0.033**
**Range**	**3–8**	**110–159**	**0.50–0.57**	**0.27–0.48**
**Lower confidence interval***	**3.29**	**116.28**	**0.505**	**0.303**
**Upper confidence interval***	**7.64**	**157.54**	**0.547**	**0.438**
**n**	**44**	**44**	**40**	**40**

SD = standard deviation
n = number of experiments
PCE: polychromatic erythrocyte
NCE:normocromatic erythrocyte
MPCE =Number of Micronucleated Polychromatic Erythrocytes referring to counts of 4000 PCE
*:The lower and upper 95% confidence intervals were calculated with C-chart.

APPENDIX IV:

CONTENTS OF THE DIET
SSNIFF® SM R/M-Z+H COMPLETE DIET FOR RATS AND MICE

Batch No.: 795 91611
Expiry Date: November 30, 2022

Crude Nutrients

Moisture (4h, 103 ºC)	13.6	%
Crude protein	20.1	%
Total fat	4.0	%
Crude fibre	4.5	%
Crude ash	6.5	%

APPENDIX V:

GLP CERTIFICATE OF TOXI-COOP ZRT

OGYÉI Országos Gyógyszerészeti és Élelmezés-egészségügyi Intézet

1135 Budapest, Szabolcs utca 33.
Levélcím: 1372 Postafiók 450
Tel.: +36 1 886 9300, Fax: +36 1 886 9460
E-mail: ogyei@ogyei.gov.hu
Web: www.ogyei.gov.hu

Ref. no: OGYÉI/21950-8/2022
Admin.: dr. Szaller Zoltán
Date: 11th August, 2022

GOOD LABORATORY PRACTICE (GLP)
CERTIFICATE

It is hereby certified that the test facility

TOXI-COOP Toxicolgical Research Center Zrt.

H-1103 Budapest, Cserkesz u. 90.,
H-1045 Budapest, Berlini u. 47-49.,
H-8230 Balatonfüred, Arácsi u. 97-99.,
H-8230 Balatonfüred, Vasút u. 3.,
H-8230 Balatonfüred, Galamb u. 12/A ,
H-8230 Balatonfüred, Ady E. u. 12,
8354 Karmacs, hrsz 4150/2

is able to carry out

physico-chemical testing, toxicity studies, mutagenicity studies, environmental toxicity studies on aquatic and terrestrial organisms, studies on behaviour in water, soil and air; bio-accumulation studies, analytical and clinical chemistry, safety pharmacology testing, metabolism and toxico/pharmacokinetics testing, testing of toxicological properties of operative procedures and equipment, reproduction toxicological studies, tolerance studies, inhalation toxicology and in vitro studies

in compliance with the Principles of GLP (Good Laboratory Practice) and also complies with the corresponding OECD/European Community requirements.
This certificate is valid till 20th of April, 2025.

Date of the inspection: **11-14 and 19-20 April, 2022.**

El Koulali Zakariás — Digitálisan aláírta: El Koulali Zakariás Dátum: 2022.08.11 15:42:41 +02'00'
Deputy Director General

APPENDIX VI:

COPY OF THE CERTIFICATE OF ANALYSIS OF THE TEST ITEM

SES research

5999 WEST 34TH STREET, SUITE 106. HOUSTON TEXAS 77092

713-686-9662
FAX 713-686-9635

Certificate of Analysis # C693

**Product Name**	ESS UTHXX in Organic Extra Virgin Olive Oil		
**Product Code:**	ESS-P411		
**Lot#**	XUTH025		
**Production Date**	04/07/2022		
**Expiration Date**	04/07/2024		
**Storage Temperature**	20-25 C, 30-70% humidity		
**Items**	**Method**	**Specification**	**Results**
Appearance	Observation	Brown Reddish liquid	PASS
Odor	Smell	Faint oil odor	PASS
Particulate Matter	USP<788>	≤ 3 particles per mL that are ≥ 25 μm in size	0 particles/mL
Carbon Fullerene	HPLC	>0.7 mg/ml	0.80 mg/ml
Fullerene blend	HPLC	4.1:1 (C60:C70)	PASS
Insoluble Impurities	≤ 0.01	≤ 0.01	≤ 0.01
Specific Gravity			
Solidify Point	volume/weight	0.909 to 0.915 at 25°F	Passed
**Heavy Metals（mg/kg or ppm）**			
Heavy Metals（mg/kg or ppm） Hg	Method EAM 4.7	<0. 010	PASS
Pb	Methods EAM 4.7	<0. 010	PASS
As	Methods EAM 4.7	0. 012	PASS
Cd	Methods EAM 4.7	<0. 010	PASS

Microbiological Specification	Quantity	Units	Method
Total Plate Count	<3000	CFU / g	USP <61>
Yeast & Mold	<300	CFU / g	AOAC
Coliforms	<3	MPN / g	FDA (BAM) Ch.4
E. Coli	Negative		USP<62>
Pseudomonas Aeruginosa	Negative		USP<62>
Salmonella	Negative		USP<62>
Staphylococcus aureus	Negative		USP<62>

SES Research

Mr. Robert Wong   04/07/2022
date

Page 1 of 1
